# Multi-step chestnut physical characteristics classification model based on vision transformation using a single-view RGB image

**DOI:** 10.1038/s41598-025-34787-6

**Published:** 2026-02-11

**Authors:** Tae Hyong Kim, Ki Hyun Kwon, Ah-Na Kim

**Affiliations:** Smart Manufacturing Research Group, Korea Food Research Institute, 245, Nongsaengmyeong-ro, Iseo-myeon, Wanju-gun, Jeollabuk-do 55365 Korea

**Keywords:** Chestnut, Vision transformer, Single-view RGB image, Computer vision, Quality assessment, Computational biology and bioinformatics, Engineering, Mathematics and computing

## Abstract

Chestnut classification is essential for improving postharvest processing efficiency and supporting large-scale commercialization; however, conventional manual sorting is labor intensive, inconsistent, and unsuitable for high-throughput operations. To address these challenges, this study proposes a k-means clustering–vision transformer (ViT)–based approach for classifying chestnuts into five cultivars, two size grades, and two rottenness states using a single-view RGB image. A total of 17,797 images were preprocessed using k-means clustering to segment chestnut regions, and four deep learning models—ViT, EfficientNetB0, ResNet-50, and DarkNet-53—were trained for multi-class classification. Model performance was evaluated using accuracy, precision, recall, and F1-score. Among the CNN models, DarkNet-53 achieved the highest performance, followed by ResNet-50 and EfficientNetB0. The ViT model outperformed all CNN models across all classification tasks, demonstrating superior pattern-recognition capability likely attributable to its self-attention mechanism, which effectively captures global contextual relationships within images. These results indicate that the proposed k-means–ViT framework provides a highly accurate and efficient solution for automated chestnut sorting. The approach shows strong potential for enhancing industrial grading systems by enabling reliable, scalable, and data-driven quality assessment.

## Introduction

Chestnut (*Castanea* spp.) is a distinctive nut crop belonging to the *Fagaceae* family. The genus *Castanea* Mill. is found in southern Europe (C. *sativa*), eastern North America (C. *dentate*), Chinese (C. *mollissima*), and Japanese and Korea (C. *crenata*). It has been planted over 2,000 years in Asian countries with precious economic and ecological functions^1^. Recently, the chestnut has become increasingly consumed important food resources due to their beneficial health effects and relatively low in calories and fat^2,3^.

It is important to provide high quality chestnuts, and an overall satisfactory experience of both fresh and processed chestnuts, in order for the consumers to become repeat customers^4^. In particular, the nuts are highly perishable nature, resulting in significant postharvest challenges. However, sorting and grading harvested chestnuts is manually conducted by operators before their refrigeration storage. The manual sorting and grading is time-consuming, labor intensive as well as generating human^5,6^. Minimum physical standards for manual operation are that they need to be, intact, sound, clean, free from pest damage, not germinated, and free from foreign tastes or smells^7^. This assessment with the naked eye is ineffective, inadequate, flawed, tedious, unpredictable, and contradictory, leading to economic loss^8^. Therefore, a more objective, rapid, and consistent method for evaluating chestnut quality is urgently needed to replace or support manual inspection, especially under industrial conditions requiring high-throughput processing. Although manual methods are still playing an important role in food processing, food industry practitioners and researchers keep working on applying innovative and emerging food processing techniques to assist food processing to reduce costs, enhance the quality of food and improve processing efficiency^9^.

In recent years, agricultural industries have started using automated systems instead of manual techniques for sorting and grading to overcome all the above shortcomings of the manual methods^10^. To assess quality or grading methods, a computer vision has been widely introduced and they h ave been proven to be scientific and useful tool. Computer vision is an engineering technology that combines mechanics, optical instrumentation, electromagnetic sensing, digital video and image processing technology^11^. The techniques have been recognized as a potential technique for the guidance or control of agricultural and food processes^12^. Recently, deep learning algorithm has been shown to be successfully used in sorting and grading process for agricultural products like fruits, vegetables, grains, even other food items such as fish, meat and dairy products using hyperspectral or spectral image^8,13^. In addition, several research applied X-ray or computer tomography image to classify or detect the physical characteristics of agricultural products using machine learning^14^. Recent studies have also demonstrated the rapid advancement of deep learning in agricultural and natural image analysis. For instance, a modified MobileNetV2 model achieved 99.8% accuracy in cashew nut and fruit disease classification, highlighting the potential of lightweight CNN architectures for nut-related image tasks^14^. Another study integrating MobileNet with an LSTM module and class-balancing techniques reported a Kappa score of 98.6% for soil-type classification, showing strong generalization performance in complex, imbalanced datasets^15^. A recent CNN-based framework also demonstrated high performance in environmental image recognition tasks^16^. These findings underscore the effectiveness of advanced deep learning models in diverse agricultural imaging applications and further support the need to evaluate robust and scalable architectures for chestnut classification. Beyond agricultural applications, deep learning has achieved remarkable success across various scientific and industrial domains, demonstrating its robustness and versatility. Recent studies have reported high-performance deep learning models in biomedical signal processing^17^, food-related quality and variety classification^18^, and advanced image-processing and computer vision tasks^19,20^. However, hyperspectral and X-ray imaging systems often suffer from slow image acquisition and processing speeds, high equipment costs, and limited suitability for high-speed industrial sorting lines. Furthermore, conventional convolutional neural networks (CNNs), although widely used for image-based sorting, primarily learn local features through convolutional kernels. This locality bias limits their ability to capture long-range dependencies and global contextual information across the entire chestnut surface. As a result, CNNs may struggle to distinguish subtle visual differences—such as fine textural patterns, cultivar-specific characteristics, or early-stage rottenness—especially when lighting conditions vary or background noise is present^21–23^. Although deep learning-based sorting and grading systems have seen significant advancements in agricultural science, many agricultural products including chestnuts have yet to be practically implemented. In particular, the high-speed operations of agricultural product processing centers pose challenges for applying deep learning techniques based on hyperspectral or X-ray imaging, due to the slower acquisition and processing speed of such systems^24^. The rapid operation required in chestnut processing facilities highlights the need for fast and cost-effective RGB-based imaging solutions. To date, no prior study has integrated a Vision Transformer with k-means clustering for multi-class chestnut classification using a single-view RGB image, offering a practical and scalable solution for high-speed industrial sorting environments.

Transformers, a type of deep neural network mainly based on the self-attention mechanism, have become the preferred models for performing Natural Language Processing (NLP) tasks (Mauricio & Domingues, 2023). Vision Transformer (ViT) is the first model to utilize transformers directly on images for object detection, without the need to integrate convolutional neural networks (CNN)^25^. ViT splits the image into several small patches, then receives each patch as input and learns the features of the image through the transformer model. Recently, interest in ViTs is increasing as a novel architecture for image recognition tasks because it gave more promising results, can outperforms CNN^26^. This is due to the its ability to learn global features, scalability, effectiveness in learning from large datasets, and the advantages of pre-training in the transformer architecture. Especially in scenarios where large amounts of data are available, ViT can more efficiently learn complex patterns compared to traditional CNNs, resulting in better performance in computer vision fields such as image classification, image segmentation, and object detection^27,28^. Mauricio et al.^29^. reported that ViTs were found to perform better and be more resilient to images with natural or adverse disturbances compared to CNNs. Deng et al.^30^. reported that the performance in the shiitake mushroom sorting reflected the ViT model’s superiority compared to CNN. Similarly, Zheng and Whang^31^ concluded that ViT models had an extensive performance evaluation for the identification of strawberry appearance quality, compared to CNN model. Importantly, the self-attention mechanism of ViT enables the model to capture global relationships across all image patches, effectively addressing the locality limitation of CNNs. This capability allows ViT to better recognize distributed and subtle patterns on agricultural products and integrate global contextual cues, suggesting its strong potential for classification tasks requiring robustness under varying imaging or environmental conditions^32,33^. Chestnut sorting requires rapid, accurate, and consistent classification, yet no practical deep learning-based system has been implemented due to high-speed processing constraints and limitations of hyperspectral/X-ray systems. Therefore, a fast and scalable RGB-based deep learning approach is essential.

In this study, we developed a k-means clustering–assisted Vision Transformer (ViT) model for multi-class chestnut classification, enabling the identification of five cultivars, two size categories, and two rottenness states using single-view RGB images. The k-means algorithm was employed to segment chestnut regions from the background prior to model training, and three widely used CNN architectures—EfficientNetB0, ResNet-50, and DarkNet-53—were additionally implemented to provide a systematic performance comparison. By evaluating accuracy, precision, recall, F1-score, and computational efficiency, this work aims to determine the most practical and robust deep learning–based strategy for real-world chestnut sorting applications. To clearly outline the novelty and scope of this work, the primary contributions of this study are summarized as follows:

- We introduce the first integrated k-means clustering–Vision Transformer framework for multi-class chestnut classification using single-view RGB images, overcoming limitations of prior hyperspectral and CNN-based approaches.
- We construct and preprocess a large-scale dataset of 17,797 chestnut images, representing five cultivars, two size grades, and two rottenness states, enabling rigorous benchmarking of deep learning architectures.
- We provide a systematic comparative analysis between ViT and three established CNN models, offering insights into their relative strengths and limitations in fine-grained agricultural image recognition.
- We demonstrate that the ViT model consistently outperforms its CNN counterparts across all classification tasks, attributable to its ability to capture global visual dependencies through self-attention mechanisms.

Following these contributions, the subsequent sections of the manuscript detail the dataset characteristics, preprocessing procedures, model architectures, and experimental protocols employed in this study, thereby establishing a clear methodological foundation for the proposed classification framework.

## Materials and methods

### Chestnut

**Fig. 1:**
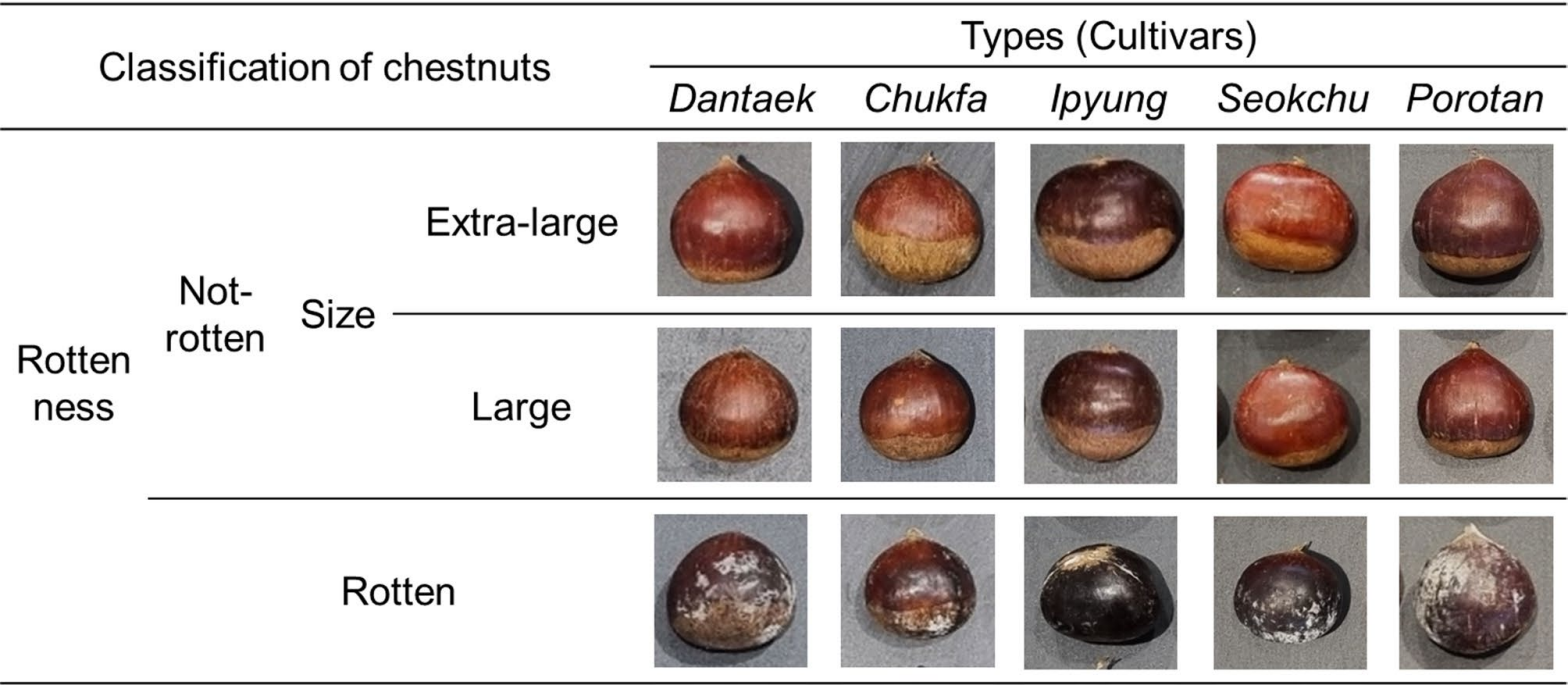
Images of chestnuts classified according to cultivars, types, and rottenness.

The chestnut five cultivars such as ‘Tanzawa (*Dantaek*)’, ‘Tsukuba (*Chukfa*)’, ‘Riheiguri (*Ipyung*)’, Ishizuchi (*Seokchu*)’, and ‘Porotan’ which were mainly produced varieties in Korea (Joo et al., 2016) were purchased from a commercial market. The fruits were harvested in Chungcheongbuk-do, Republic of Korea in September 2023. They were washed and selected right after harvesting, and then stored at 1 ± 0.5°C in the dark before use. The chestnuts were classified and sorted according to cultivars, sizes, and rottenness (Fig. 1). The chestnuts are divided into five classes of different cultivars (*Dantaek*, *Chukfa*, *Ipyung*, *Seokchu*, and Porotan), two classes of sizes (Extra-large and Large), and two classes of rottenness (Not rotten and Rotten).

### Image data acquisition

The red-green-blue (RGB) image data of chestnut after postharvest were acquired. Raw chestnuts were arranged and placed one by one on a plate which was coated with matte black in order to minimize the effect of reflected lighting. Thereafter, image data were acquired using a RGB camera which is installed at the fixed top. The fixing jig of RGB camera is located 40 cm away from the plate in order to collect the image data constant distance to minimize calibration error. The size of raw RGB image of the chestnut are 4000 × 3000 × 3 pixel. Raw images are captured by connecting GoPro cameras (Hero 10, California, USA) with android phone by wireless fidelity (GoPro Quik). The captured raw images were stored in secure digital (SD) card for future image processing and classification.

### Data preprocessing

**Fig. 2:**
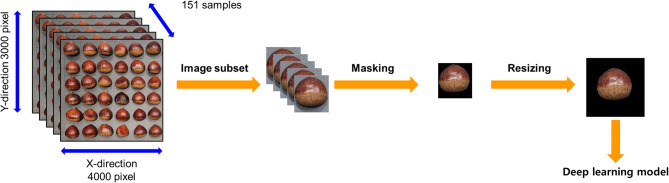
Data preprocessing to remove background and match image size of acquired raw RGB image data of chestnut.

The first step in preprocessing the acquired raw RGB images of chestnuts is subsetting each individual chestnut, as illustrated in Fig. 2. Depending on the weight of chestnut batch while storing in cold room, the number of chestnut in each batch are different. Chestnut batch image are arranged and labeled in order by original chestnut batch images. Total of 17,797 single-view RGB chestnut subset image are extracted.

In order to remove background from original image to improve the performance of classification of chestnut, the background removal process is processed by first apply median filter to remove noise in RGB images. To remove background, image segmentation process is applied. The process of removing the background from the original image using clustering algorithm is described as follow. First, a filter RGB image is transform into L*A*B color space to apply as input image for *k*-means clustering algorithm. The *k*-means clustering algorithm which is one of representative unsupervised clustering algorithm. It first selects number of cluster, set the initial centroid, calculate the distance for input data from initial centroid and determine new centroid point. This step is calculating iteratively until there is no change of centroid to segment similar pixel value. Iteratively calculate centroid point until is applied to divide background and objects in filter images. K-value for *k*-means clustering algorithm is 2 in this study with number of iteration of 50. The extracted image from k-means clustering algorithm then used as input to active contour algorithm for final mask image. The mask image values are composed of [0, 1] where value 0 is background region and 1 is chestnut region. From the masking image, the pixels with value 1 is replaced with original RGB pixel value. Last step of single-view RGB image preprocessing is to match the image size. First is to create empty 3D matrix with size of 224 by 224 by 3. Each subset back-ground-removed image is then placed on the center of empty 3D matrix to match each subset image.

**Fig. 3:**
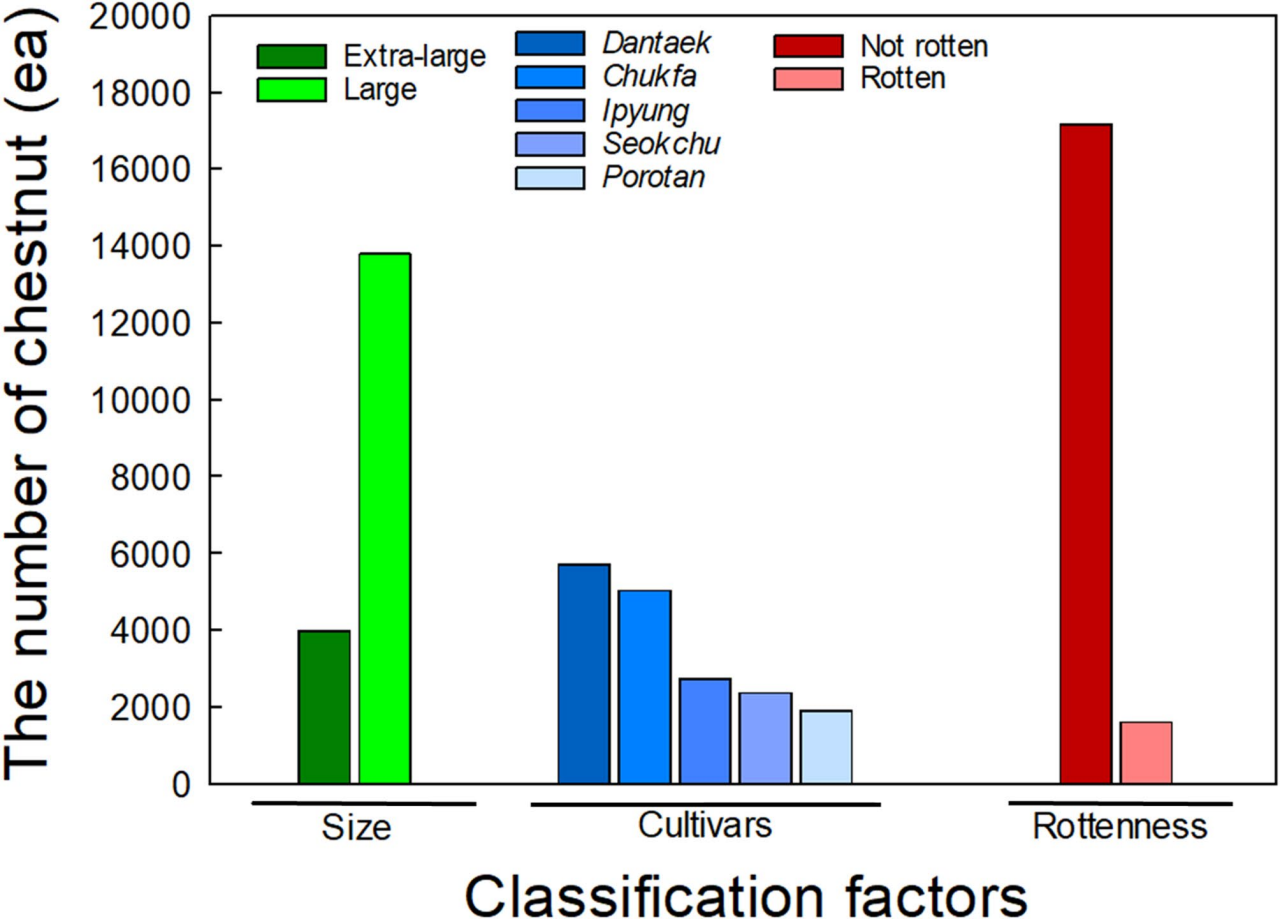
Distribution charts for (**A**) cultivar, (**B**) size, and (**C**) rottenness of chestnuts labelled from acquired images.

In this study, the development of multi-class classification model using deep learning to classifying type of chestnut, size of chestnut and rot/decay status of chestnut requires labeling of each images. Three people labeled each subset image for chestnut type classification model. There are 5 classes of chestnut type with the number of each cultivar. Next, there are two classes for chestnut size and rot/decay status, respectively. After labeling process finishes, we calculated the frequency of each image labeled class and select the most frequent class type for cultivar, size and rottenness to minimize human labeling error. Chestnut clustered images are manually labeled using MATrix LABoratory(MATLAB) Image Labeler tool (version 2023b, Mathworks, Natick, MA, USA, https://www.mathworks.com). Distribution of chestnuts images labeled according to five cultivars, two size, and rotten or not are shown in Fig. 3. The distribution of RGB image for two class sizes are 3993 (Extra-large) and 13,803 (Large), respectively. In addition, distribution of RGB image for rottenness classification are 17,177 (Not rotten) and 620 (Rotten), respectively. Last, the distribution of cultivar classifications is 5713 (Dantaek), 5046 (Chukfa), 2740 (Ipyung), 2380 (Seokchu) and 1919 (Porotan), respectively with total of 17,797 image.

### Deep learning model

Previous researches applied to deep learning model using image data for categorize or classify the illness of food, detecting sprout of potatoes^34,35^. Detecting the specific class or type or its condition of food, deep convolutional neural network (CNN) model shows amazing performance as compare to traditional machine learning models.

**Fig. 4:**
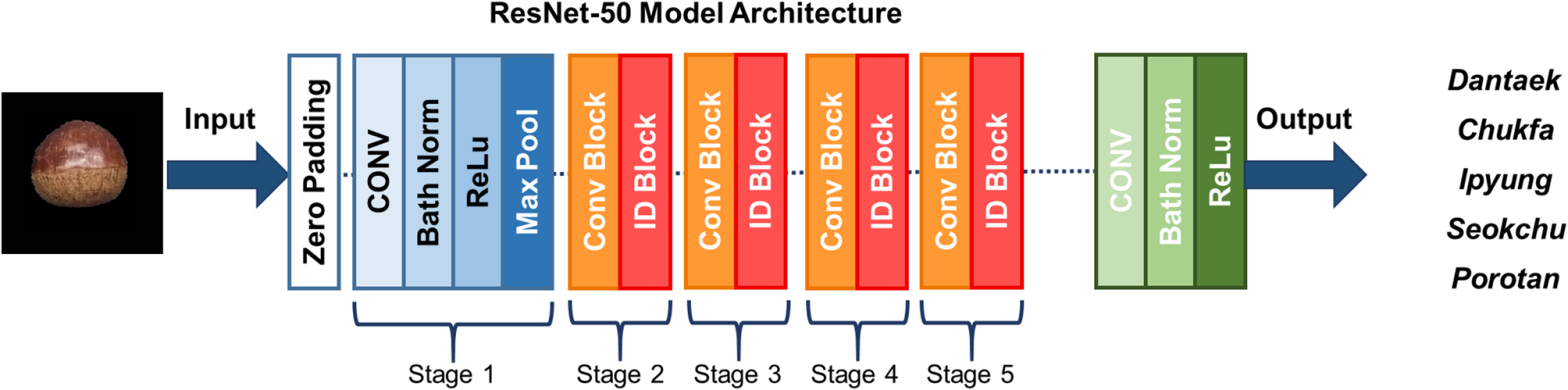
Representative ResNet-50 deep CNN architecture.

There are several image classification CNN deep learning model architecture such as SqueezeNet or AlexNet^36^. In this study, three multi-class deep learning models for chestnut physical characteristics are developed. Each deep learning model classify chestnut type, size, and rottenness. Three CNN architectures were implemented together with the ViT model for each multi-class classification task. To ensure scientific rigor and methodological transparency, the selection of the four deep learning architectures used in this study—EfficientNetB0, ResNet-50, DarkNet-53, and the Vision Transformer (ViT)—was based on their complementary strengths and their suitability for the objectives of chestnut classification. EfficientNetB0 was chosen as a lightweight CNN with strong computational efficiency, providing a baseline for evaluating performance under real-time constraints^37^. ResNet-50 was included because its residual learning framework mitigates vanishing gradients in deep architectures, offering stable representation learning^38^. DarkNet-53, originally designed for high-speed object detection in the YOLOv3 architecture, was selected for its deeper feature extraction capability and fast inference, characteristics advantageous for industrial sorting environments^39^. Finally, ViT was incorporated due to its ability to capture long-range dependencies and global contextual information through self-attention, making it particularly effective for distinguishing subtle variations in chestnut surface patterns, cultivar traits, or early-stage rottenness^27^. Together, these models allow a comprehensive assessment of accuracy, efficiency, and robustness across different deep learning paradigms. One of the famous CNN architecture which is ResNet-50 applied residual learning method to stack multiple of layers. In usual case, deep learning model performance increase where number of layer increases. However, by stacking layers, gradient vanishing and gradient exploding appears where the performance degrades. As mentioned above, ResNet architecture applied residual learning method which add x value of input of convolutional layer to output of convolutional layer which train only difference of input and output value. The ResNet-50 composed of 50 layers as shown in figure below (Fig. 4)^40^. To implement ResNet-50 architecture to classify chestnut type, size, and rottenness, each classification model’s classification layer is replaced and applied transfer learning technique to freeze layers.

**Fig. 5:**
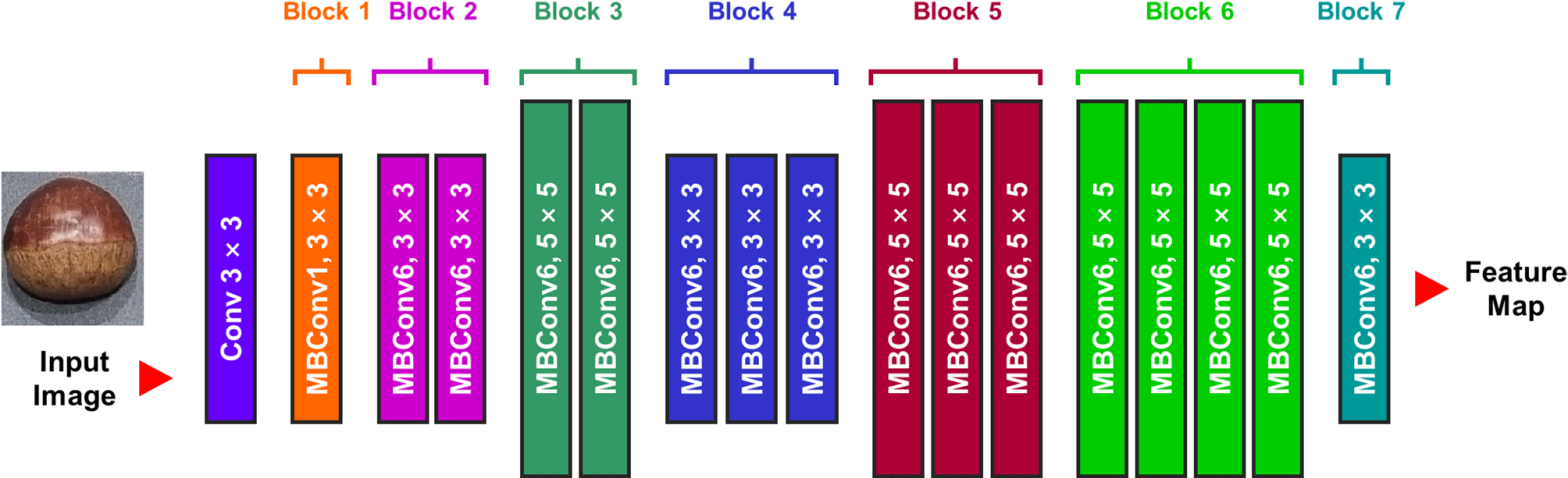
Representative EfficientnetB0 deep CNN architecture.

Next deep learning architecture applied in this study is EfficientNet. The advantage of EfficientNet is that the small number of parameter of layers, the performance is relatively higher than other deep learning architectures. To increase the size of architecture, there are three factors to consider. Depth, width and resolution size can be increased. The major concept of EfficientNet is to determine a, b,r value of depth, width and resolution. In this study, EfficientNet-b0 model which composed of 5.3 M parameters is applied for classification (Fig. 5)^41^. Replacement of last classification layer with transfer learning is implemented as similar to ResNet-50.

**Fig. 6:**
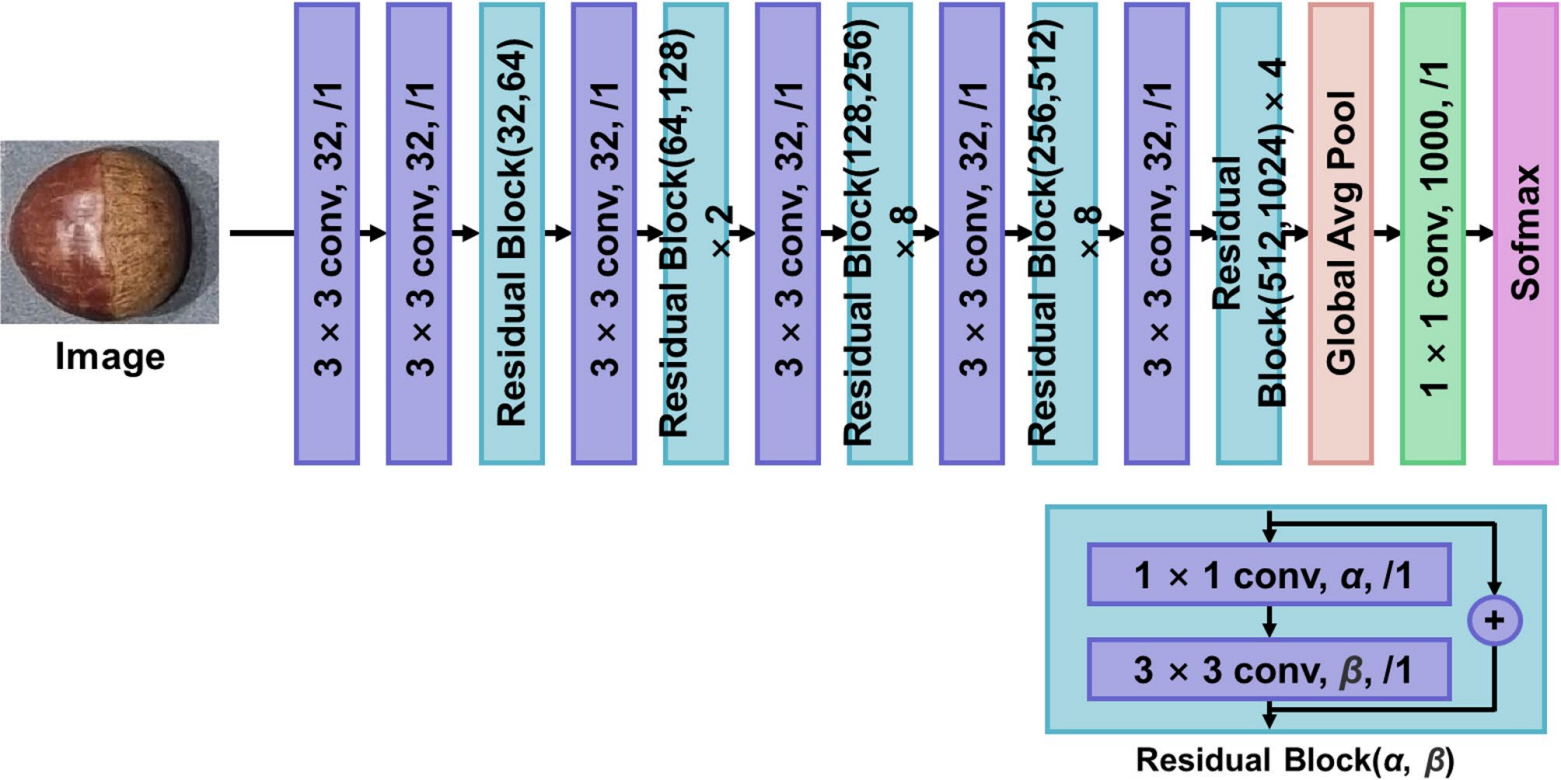
Representative Darknet-53 deep CNN architecture.

Third deep learning architecture applied in this study is DarkNet which is developed for object detection. DarkNet53 applied similar concept of residual network of ResNet architecture by repeatedly add 3 × 3 and 1 × 1 convolutional layer and connecting shortcut up to 53 convolution layer (Fig. 6)^42^.

**Fig. 7:**
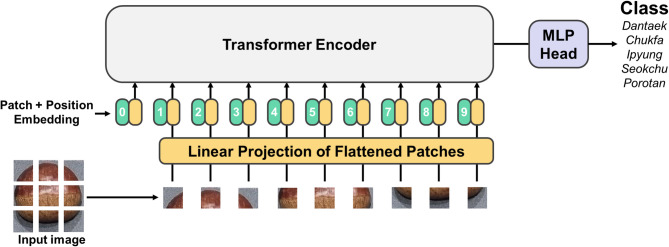
Representative ViT architecture.

**Fig. 8:**
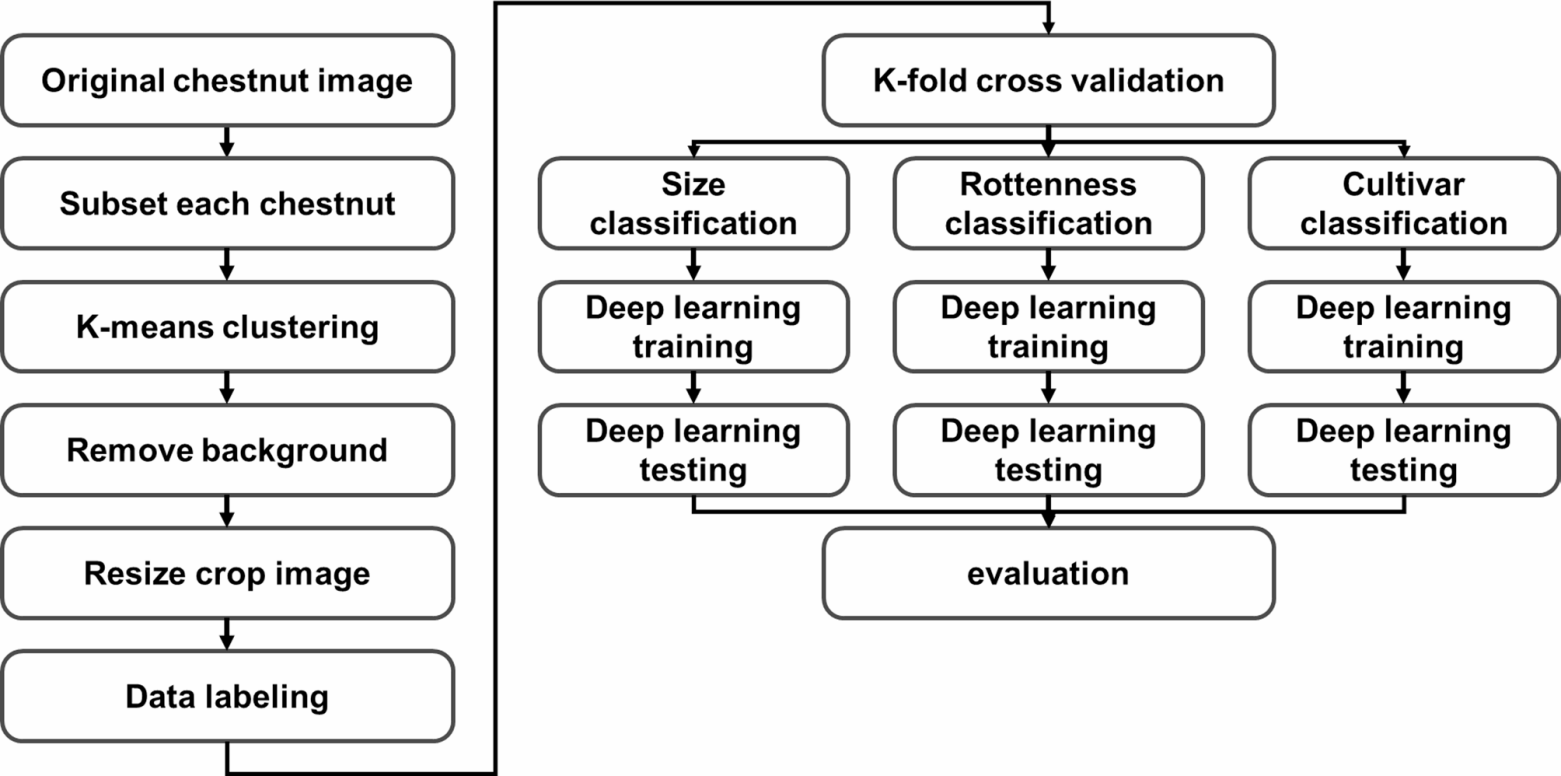
Overall flowchart of k-means deep learning based chestnut physical characteristic classification model.

Lastly, vision transformer (ViT) which is developed for natural language processing shows outstanding performance for computer vision by applying transformer in image classification or object detection research field. Especially, ViT is widely used for extracting feature from image. Similar to deep learning CNN, ViT is a supervised learning training model with high computational efficiency and scalability. ViT works by inserting image to standard transformer with preprocessing of image by divide image into path unit to be tokenized. Each image path is flattened to transform into vector, and linear projection calculation is processed for embedding task. After transformer encoder, output of token is than used as input of MLP for classification (Fig. 7)^43^. To implement ViT (base-16-imagenet-384 model) to classify class type, size and rottenness, the classification layer for each model is replaced to 5, 2 and 2. In addition, to fine tune ViT model, training options with learning rate of 0.001, momentum of 0.9 with cross entropy loss function, batch size of 32, number of 1,000, learning rate schedule of piecewise is implemented. While transfer learning, we freeze patch embedding and transformer block of 1–9 and train transformer block of 10–12 and classification head which is standard transfer learning technique. The overall flow of image-based deep learning model classification of size, rottenness and cultivar for chestnut is shown in Fig. 8.

### Performance evaluation

To create the image dataset to use as training deep learning model, the RGB chestnut image dataset is divided into training and test group with the ratio of 80:20, respectively to apply 5-fold cross validation while training and testing deep learning model.

In order to evaluate each cultivar, size, and rottenness classification performance of a model, some evaluation metrics have been introduced and calculated. In this research used four performance evaluation parameters: accuracy (1), macro-precision (2), macro-recall (3), and micro-F1-score (4) based on the confusion matrix. Accuracy is the ratio of correctly predicted instances to the total instances. Precision is the ratio of correctly predicted instances for a specific class to all instances predicted as that class. Recall (sensitivity) is the ratio of correctly predicted instances of a class to the actual number of instances in that class. F1 score is The harmonic mean of precision and recall, providing a balance between the two^44^. Equations for four parameters are shown as follows:

1 $$\:\mathrm{A}\mathrm{c}\mathrm{c}\mathrm{u}\mathrm{r}\mathrm{a}\mathrm{c}\mathrm{y}\:\left(\mathrm{\%}\right)=\:\frac{\mathrm{N}\mathrm{u}\mathrm{m}\mathrm{b}\mathrm{e}\mathrm{r}\:\mathrm{o}\mathrm{f}\:\mathrm{c}\mathrm{o}\mathrm{r}\mathrm{r}\mathrm{e}\mathrm{c}\mathrm{t}\mathrm{l}\mathrm{y}\:\mathrm{c}\mathrm{l}\mathrm{a}\mathrm{s}\mathrm{s}\mathrm{i}\mathrm{f}\mathrm{i}\mathrm{e}\mathrm{d}\:\mathrm{c}\mathrm{l}\mathrm{a}\mathrm{s}\mathrm{s}\mathrm{e}\mathrm{s}\:}{\mathrm{T}\mathrm{o}\mathrm{t}\mathrm{a}\mathrm{l}\:\mathrm{n}\mathrm{u}\mathrm{m}\mathrm{b}\mathrm{e}\mathrm{r}\:\mathrm{o}\mathrm{f}\:\mathrm{s}\mathrm{a}\mathrm{m}\mathrm{p}\mathrm{l}\mathrm{e}\mathrm{s}}\times\:100$$

2 $$\:\mathrm{P}\mathrm{r}\mathrm{e}\mathrm{c}\mathrm{i}\mathrm{s}\mathrm{i}\mathrm{o}\mathrm{n}\:\left(\mathrm{\%}\right)=\:\frac{\mathrm{T}\mathrm{r}\mathrm{u}\mathrm{e}\:\mathrm{P}\mathrm{o}\mathrm{s}\mathrm{i}\mathrm{t}\mathrm{i}\mathrm{v}\mathrm{e}}{\mathrm{T}\mathrm{r}\mathrm{u}\mathrm{e}\:\mathrm{P}\mathrm{o}\mathrm{s}\mathrm{i}\mathrm{t}\mathrm{i}\mathrm{v}\mathrm{e}+\mathrm{F}\mathrm{a}\mathrm{l}\mathrm{s}\mathrm{e}\:\mathrm{P}\mathrm{o}\mathrm{s}\mathrm{i}\mathrm{t}\mathrm{i}\mathrm{v}\mathrm{e}}\times\:100$$

3 $$\:\mathrm{R}\mathrm{e}\mathrm{c}\mathrm{a}\mathrm{l}\mathrm{l}\:\left(\mathrm{\%}\right)=\:\frac{\mathrm{T}\mathrm{r}\mathrm{u}\mathrm{e}\:\mathrm{P}\mathrm{o}\mathrm{s}\mathrm{i}\mathrm{t}\mathrm{i}\mathrm{v}\mathrm{e}}{\mathrm{T}\mathrm{r}\mathrm{u}\mathrm{e}\:\mathrm{P}\mathrm{o}\mathrm{s}\mathrm{i}\mathrm{t}\mathrm{i}\mathrm{v}\mathrm{e}+\mathrm{F}\mathrm{a}\mathrm{l}\mathrm{s}\mathrm{e}\:\mathrm{N}\mathrm{e}\mathrm{g}\mathrm{a}\mathrm{t}\mathrm{i}\mathrm{v}\mathrm{e}}\times\:100$$

4 $$\:\mathrm{F}1\:\mathrm{s}\mathrm{c}\mathrm{o}\mathrm{r}\mathrm{e}=\:\frac{\mathrm{P}\mathrm{r}\mathrm{e}\mathrm{c}\mathrm{i}\mathrm{s}\mathrm{i}\mathrm{o}\mathrm{n}\:\times\:\mathrm{R}\mathrm{e}\mathrm{c}\mathrm{a}\mathrm{l}\mathrm{l}}{\mathrm{P}\mathrm{r}\mathrm{e}\mathrm{c}\mathrm{i}\mathrm{s}\mathrm{i}\mathrm{o}\mathrm{n}+\mathrm{R}\mathrm{e}\mathrm{c}\mathrm{a}\mathrm{l}\mathrm{l}}\times\:2$$

The experimental system type used was Windows 10 Pro 64-bit operating system. The processor was Intel Core i9-13900HX, using GPU NVIDIA A4500, 24 GB graphics card. The software environment was supported by MATLAB computer vision toolbox and deep learning toolbox (version 2023b, Mathworks, Natick, MA, USA, https://www.mathworks.com).

## Results and discussion

The proposed single-view RGB image-based multi-step chestnut classification models are designed using deep learning architectures combined with the k-means image segmentation clustering algorithm. The chestnut sample image datasets were acquired using RGB cameras. The dataset contains frontal view images of 17,797. In this dataset, 5-fold cross validation technique is used to divide training, validation and test dataset. The 14,238 segmented images are input into the proposed model for training and rest of 3,559 images are used to test results. To determine the cultivar, size, and rottenness classification performance using deep network, we conducted a comparative study analysis among four different models including EfficientnetB0, ResNet-50, DarkNet-53, and ViT.

### Cultivar classification model

The chestnut quality (taste, sugar content, and shell thickness) varies depending on the cultivar of chestnut, and it is important to distinguish the types of chestnuts because it is necessary to select the right use, manage the shelf life, differentiate the price, and select a processing condition suitable for process suitability^40,45^.

**Fig. 9:**
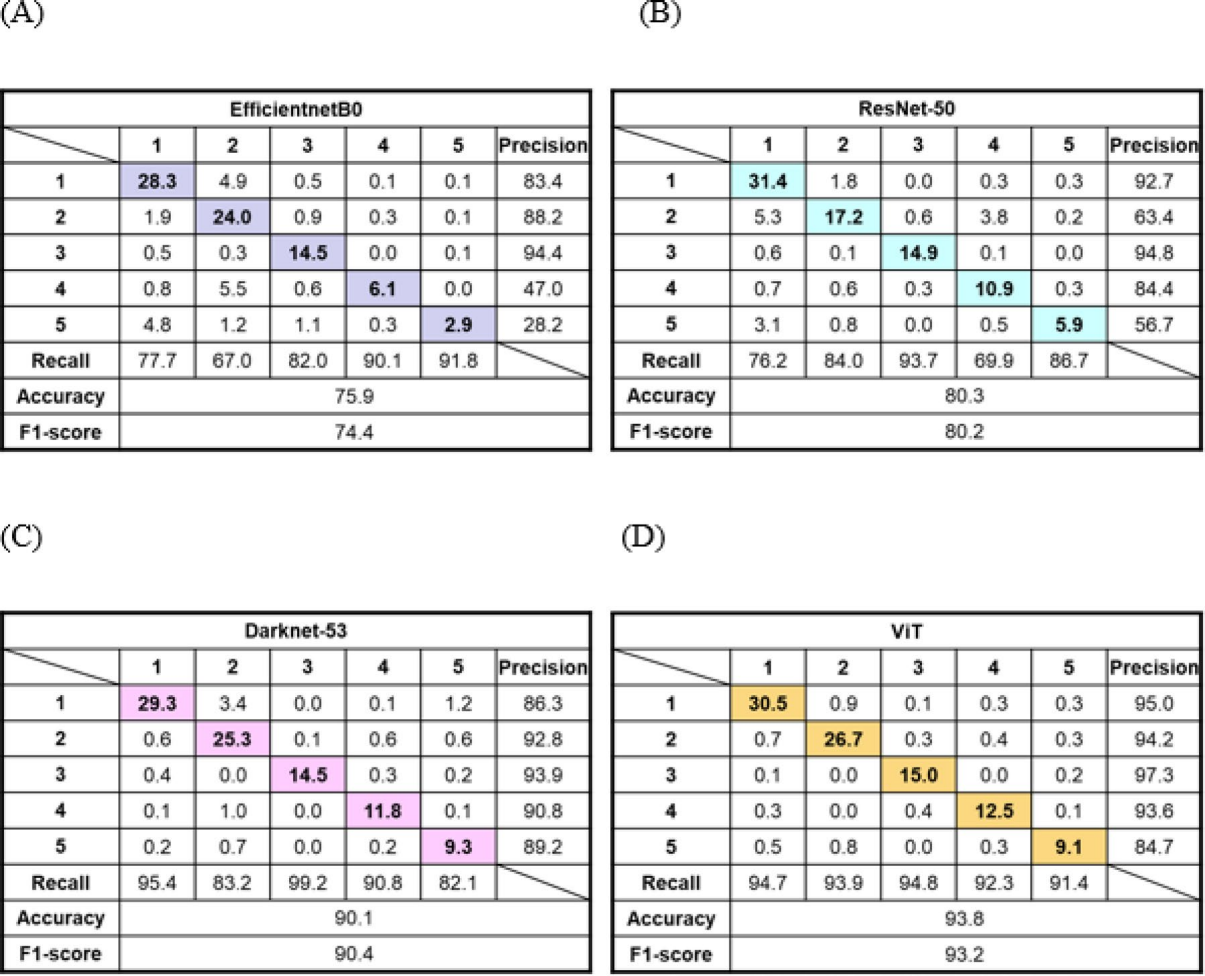
Representative confusion matrices of chestnut cultivar classification implementing (**A**) EfficientnetB0, (**B**) ResNet-50, (**C**) Darknet-53, and (**D**) ViT model. All values in the confusion matrices are expressed as percentages (%). Colored cells represent correctly classification.

The performance evaluation parameters of proposed four models for chestnut cultivar classification are shown in Table 1. The ViT showed the best overall performance in chestnut cultivar classification. In detail, the ranking of all parameters is ViT > Darknet-53 > Resnet-50 > EfficientnetB0 with showing a big difference among the models. The performance evaluation parameters such as accuracy, recall, precision, and F1-score of ViT model are 1.27, 1.15, 1.37, and 1.26 times higher than EfficientnetB0 which had the lowest all parameters. Figure 9 presents a representative confusion matrices of cultivar classification for four deep learning models. The cultivar classes 1, 2, 3, 4, and 5 indicate *Dantaek*, *Chukfa*, *Ipyung*, *Seokchu*, and Porotan, respectively. The number of predicted and measured values was the most consistent in ViT model in accordance with the result of performance evaluation parameters. Among the models evaluated, the ViT model demonstrated the highest performance in classifying chestnut cultivars, with the Darknet-53 model ranking second.

**Table 1:** Performance evaluation parameters of four chestnut cultivar classification models. ViT represents vision transformer.

Classification	Model types	Performance parameters			
		Accuracy (%)	Recall (%)	Precision (%)	F1-score
Cultivar	EfficientnetB0	73.5 ± 2.4	81.3 ± 0.4	67.9 ± 0.4	74.0 ± 0.4 (*p* = 4.7 × 10⁻¹¹)
	ResNet-50	79.0 ± 1.3	82.1 ± 0.0	76.4 ± 2.0	79.1 ± 1.1 (*p* < 1.0 × 10⁻⁶)
	Darknet-53	90.1 ± 0.0	90.0 ± 0.1	90.6 ± 0.0	90.3 ± 0.1 (*p* = 1.9 × 10⁻⁵)
	ViT	93.3 ± 0.5	93.4 ± 0.5	92.9 ± 0.0	92.9 ± 0.3 (reference)

Statistical analysis was performed using a two-sided paired t-test to compare F1-scores of CNN-based models with that of the ViT model across 5-fold cross-validation. Differences were considered statistically significant at *p* < 0.05.

### Size classification model

**Fig. 10:**
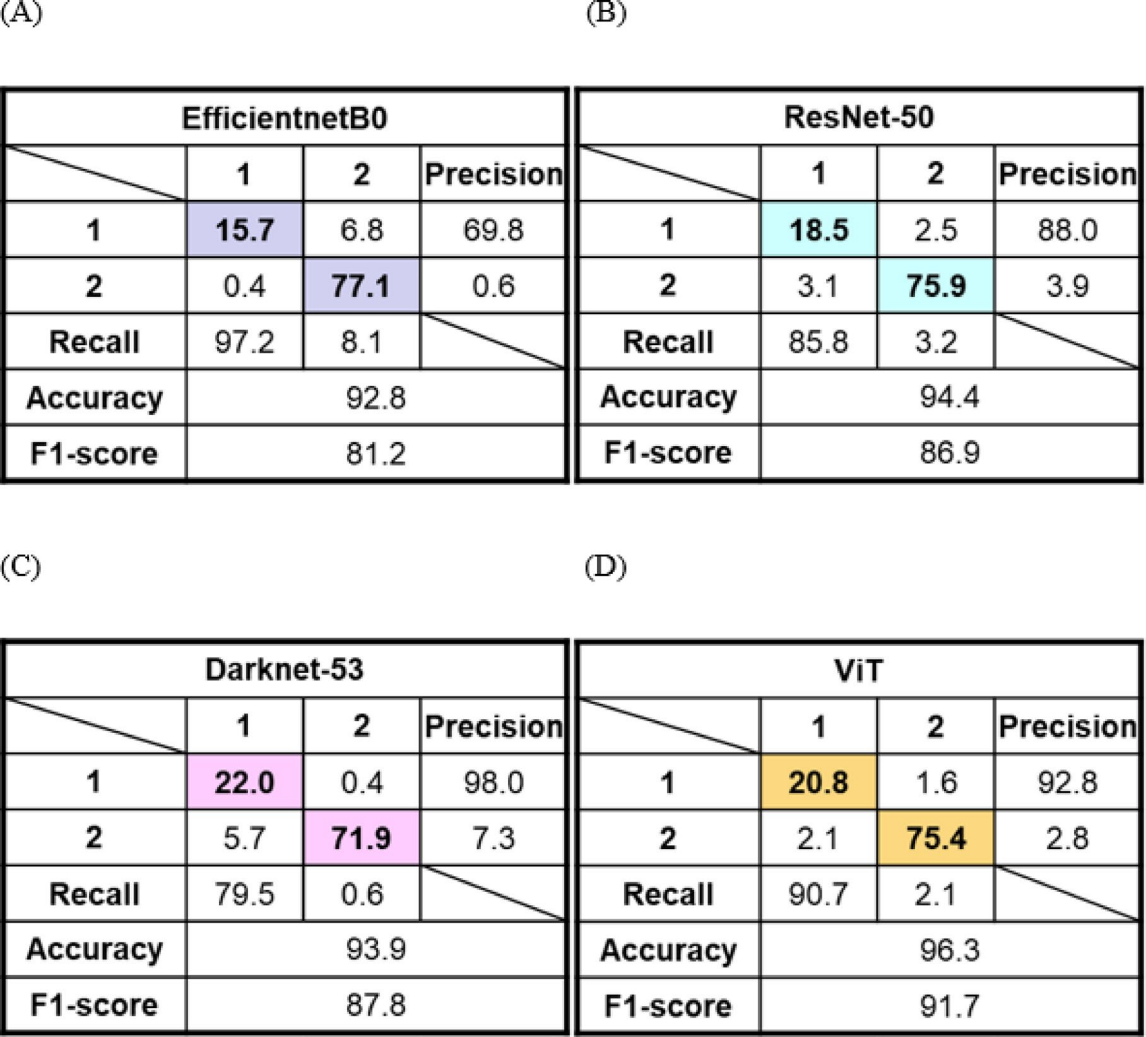
Representative confusion matrices of chestnut size classification implementing (**A**) EfficientnetB0, (**B**) ResNet-50, (**C**) Darknet-53, and (**D**) ViT models. All values in the confusion matrices are expressed as percentages (%). Colored cells represent correctly classification.

The chestnuts were usually sorted by a chestnut size separator^46^. The centrally located cylindrical drum of the separator have holes of various sizes. As the drum rotates, the chestnuts fall down through each hole depending on their size. However, there is a problem of misclassification of chestnut size when they rotate in drum because the chestnut is not perfectly spherical. In this study, four deep learning models for chestnut size classification were proposed, and then their performance was evaluated (Table 2). The chestnuts were graded as two sizes of large (class 1) and extra-large (class 2) in Fig. 10. While EfficientnetB0 model achieved the highest value for recall, it recorded the lowest values across the remaining three parameters. This imbalance between precision and recall led to the lowest value observed for F1-score. On the other hand, ViT model achieved the highest values for the accuracy, precision, and F1-score, and exhibited the second-highest. As a result of considering all parameters, ViT model performed best for chestnut size classification, followed by the DarkNet-53 model.

**Table 2:** Performance evaluation parameters of four chestnut cultivar classification models. ViT represents vision transformer.

Classification	Model types	Performance parameters			
		Accuracy (%)	Recall (%)	Precision (%)	F1-score
Size	EfficientnetB0	92.9 ± 0.1	95.8 ± 1.4	70.8 ± 1.1	81.4 ± 0.2 (*p* = 8.6 × 10⁻¹³)
	ResNet-50	93.5 ± 0.9	79.2 ± 6.6	93.3 ± 5.3	85.2 ± 1.6 (*p* = 3.4 × 10⁻⁴)
	Darknet-53	94.1 ± 0.2	84.7 ± 5.2	91.2 ± 6.8	87.4 ± 0.4 (*p* = 6.2 × 10⁻⁶)
	ViT	96.5 ± 0.2	91.6 ± 0.9	92.9 ± 0.1	92.2 ± 0.5 (reference)

Statistical analysis was performed using a two-sided paired t-test to compare F1-scores of CNN-based models with that of the ViT model across 5-fold cross-validation. Differences were considered statistically significant at *p* < 0.05.

### Rottenness classification model

**Fig. 11:**
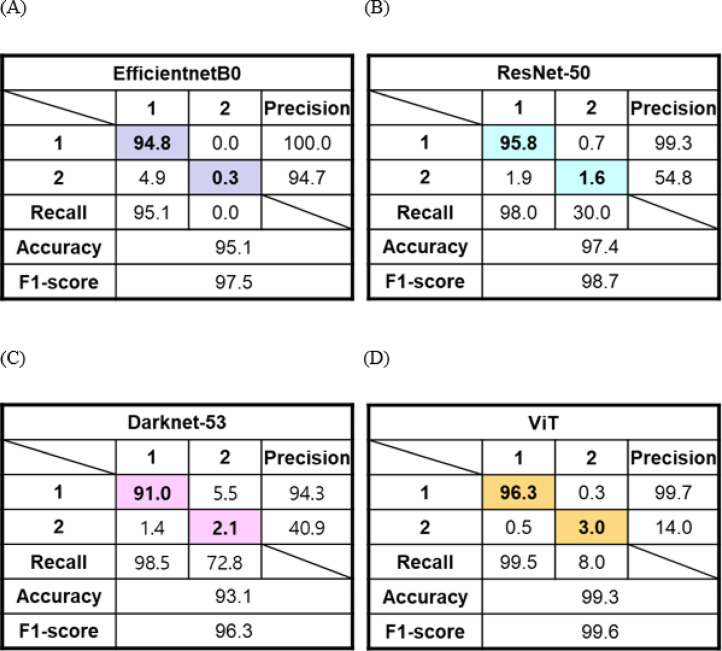
Representative confusion matrices of chestnut rottenness classification implementing (**a**) EfficientnetB0, (**b**) ResNet-50, (**c**) Darknet-53, and (**d**) ViT models. All values in the confusion matrices are expressed as percentages (%). Colored cells represent correctly classification.

The chestnut is highly perishable and poses significant food postharvest challenges^46^. Several types of rotten chestnuts result from microbial growth, including bacteria, yeasts, and molds, as well as physical damage such as wormholes, cracks, and scratches, leading to zero commercial value^47^. Furthermore, rotten chestnut accelerates reduction of shelf life because it can spread of microbial contamination and increases humidity by releasing moisture as they decompose when stored together with healthy chestnuts^47–51^. In general sorting process, floating chestnut during washing process is regarded as rotten chestnut due to decreased density caused by deterioration of internal structure, and removed. However, it cannot sort the other rotten chestnut, except for the most severe decay. The performance evaluation parameters of proposed four deep learning models for rottenness classification was indicated in Table 3. All parameters such as accuracy, macro-recall, macro-precision, and F1-score had high values ranged from 93.4% to 99.3%, 95.3% to 99.5%, 96.6% to 100%, and 97.6 to 99.6, for all models, respectively. Among them, ViT model indicates the best performance by indicating the highest values of accuracy, macro-recall, and F1-score. Figure 11 showed the representative confusion matrix for classification of rotten (class 2) or not (class 1). Classification of rotten chestnuts had very good performance, but even not-rotten chestnuts were often misclassified as rotten in EfficientnetB0 model. On the other hand, the number of matching predicted and measured values for both rotten and not rotten in ViT model was high. Consequently, the model performance was best evaluated for sorting rotten or not chestnut in order of ViT, Darknet-53, ResNet-50, and EfficientnetB0.

**Table 3:** Performance evaluation parameters of four chestnut rottenness classification models. ViT represents vision transformer.

Classification	Model types	Performance parameters			
		Accuracy (%)	Recall (%)	Precision (%)	F1-score
Rottenness	EfficientnetB0	95.3 ± 0.2	95.3 ± 0.2	100.0 ± 0.0	97.6 ± 0.1 (*p* = 7.2 × 10⁻¹⁶)
	ResNet-50	97.8 ± 0.4	98.8 ± 0.7	99.0 ± 0.3	98.8 ± 0.2 (*p* = 1.6 × 10⁻⁶)
	Darknet-53	93.4 ± 0.4	98.5 ± 0.0	96.6 ± 0.3	99.3 ± 0.0 (*p* = 3.9 × 10⁻⁵)
	ViT	99.3 ± 0.0	99.5 ± 0.0	99.7 ± 0.0	99.6 ± 0.0 (reference)

Statistical analysis was performed using a two-sided paired t-test to compare F1-scores of CNN-based models with that of the ViT model across 5-fold cross-validation. Differences were considered statistically significant at *p* < 0.05.

### Deep learning training performance

**Fig. 12:**
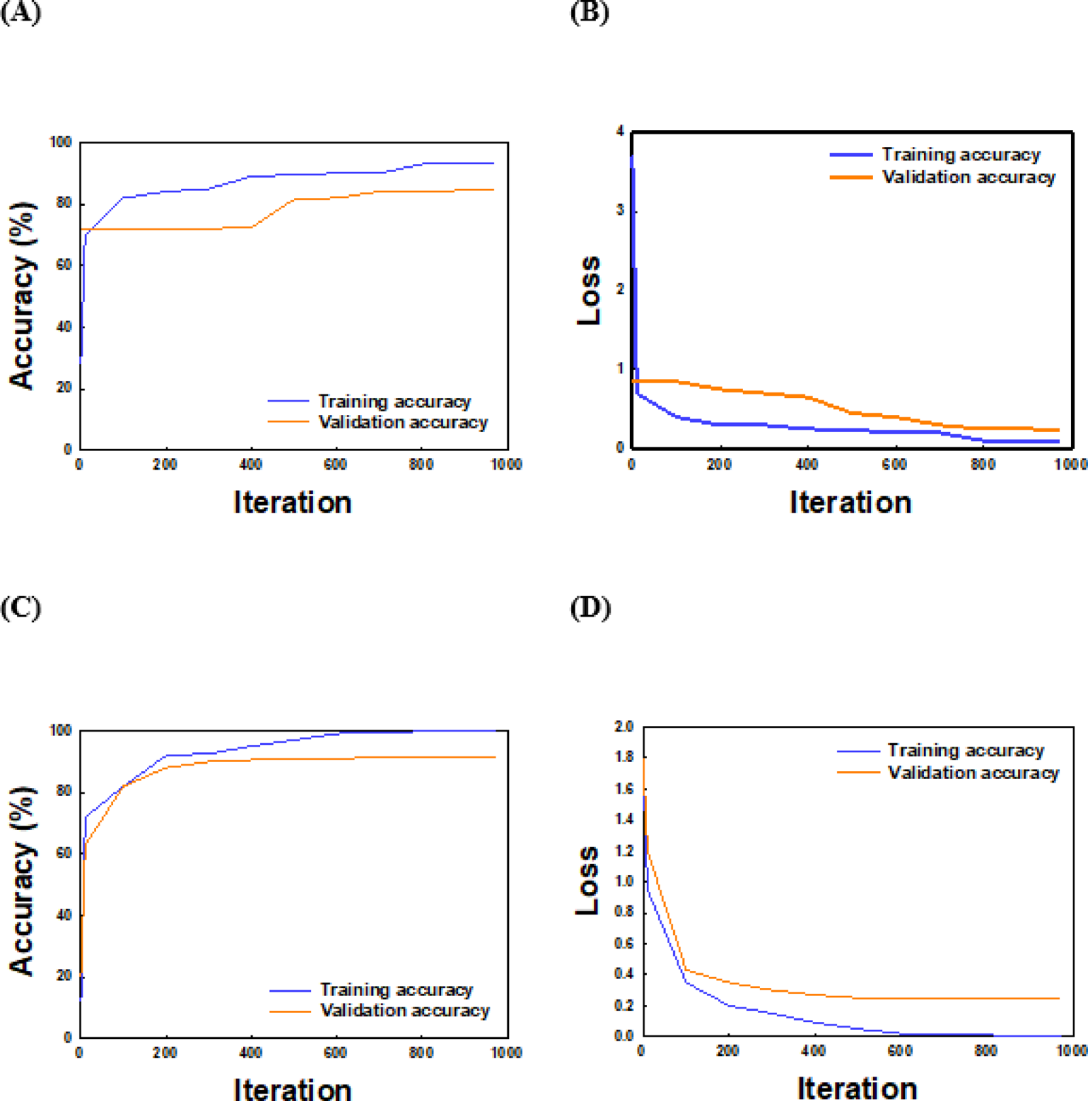
Accuracy and loss curves of training and validation accuracy of Darknet-53 and ViT model classification performance depends on training epochs and optimization generation. Left figure represents the training accuracy and right figure represents loss results.

Validation set for accuracy value and loss value during training of DarkNet-53 and ViT model were obtained according to iteration respectively as shown in Fig. 12. The loss curve of ViT model (Fig. 12b) decreased rapidly during training and the training time was short, which could well meet the actual sorting needs, compared to DarkNet-53 model (Fig. 12d).

In cultivar, size and rottenness multi-class classification performance result, ViT model demonstrated superior performance compared to deep CNN models. In this study, among the three deep CNN models, the EfficientnetB0 showed lowest performance even with higher CNN layer architecture as compare to ResNet-50. In addition, ResNet-50 performance was lower than DarkNet-53 with similar number of CNN layer. Even with similar number of layer, the performance varies due to the number of training parameter is different. For instance, DarkNet-53 composed of 41.6 million training parameter as compare to EfficientnetB0 composed of 5.3 million training parameters (Table 4). The computational FPS for EfficientnetB0, ResNet-50, Darkent-53 and ViT model showed 162.95, 132.77, 86.65 and 40.75, respectively. In addition, the ViT classification performance is higher than rest of 3 deep CNN model. This might due to the difference of architecture design where ViT utilize self-attention mechanism.

**Table 4:** Computational performance of each deep learning model for testing rottenness classification model.

Classification	Model types	FPS	Number of parameter	Model size (Mb)
Rottenness	EfficientnetB0	162.95	5.3 Millions	20
	ResNet-50	132.77	25.6 Millions	98
	Darknet-53	86.65	41.6 Millions	159
	ViT	40.75	86.8 Millions	331

Computer vision in food processing enable the objective monitoring and control of various quality parameters such as size, shape, texture, and color which minimize human error from repetitive tasks^52^. Among deep learning approaches, CNN is recognized as key methods in the field of image analysis due to its multi-layered and carefully trained architecture. Since 2015, deep CNNs have been increasingly applied in the agricultural and food sectors, primarily for object recognition and classification^53^. VijayaKumari et al.^54^. employed EfficientNetB0 model on the Food-101 dataset and achieved approximately 80% accuracy, demonstrating its effectiveness in food image classification tasks with limited data. However, applying CNNs to large-scale food image classification remains difficult due to the high intra-class similarity and complex inter-class variability commonly observed in such datasets. These factors significantly hinder classification performance, indicating that further advancements^55^. The ViT, proposed by Dosovitskiy et al.^27^. , effectively addresses the challenges of category diversity and high shape similarity by utilizing a self-attention mechanism on image patches to capture global representations. Furthermore, semi-supervised learning enables ViT to leverage unlabeled data, thereby enhancing its performance and generalization ability^56^. Mauricio et al.^29^. reported that the ViT architecture is more robust than CNNs when dealing with noisy or augmented images, thanks to its self-attention mechanism that allows access to global image information across all layers. While CNNs tend to generalize better and achieve higher accuracy on smaller datasets, ViTs can learn more effectively from fewer images by dividing them into patches, which enables learning a wider range of relationships. The ViTs outperform CNNs in food image recognition by capturing global dependencies through self-attention, enabling better differentiation of visually similar categories^57^. Their flexibility, transferability, and robustness to visual variability make them well-suited for complex datasets. Gao et al.^55^. reported that ViT achieves superior performance across accuracy, precision, recall, and F1-score, demonstrating both strong fitting ability and robust generalization to unseen data in food image classification tasks. Importantly, when compared with previously reported agricultural and food image classification studies, the performance achieved in this work shows clear improvements. Prior CNN-based models such as EfficientNet, MobileNetV2, and other lightweight architectures typically reported accuracies in the range of 80–95% on nut, fruit disease, soil-type, and environmental datasets^14,15,54^. These results demonstrate the potential of CNNs but also highlight their limitations in handling high intra-class similarity and subtle quality defects—problems that are also common in chestnut classification. In contrast, the ViT model in this study achieved substantially higher accuracy, precision, recall, and F1-score across all classification categories (cultivar, size, and rottenness). This observation is consistent with the findings of recent ViT-based agricultural research, which report superior robustness and generalization ability when compared with CNNs^29,55,57^. Therefore, these comparisons reinforce that the proposed k-means + ViT framework provides a more scalable and accurate approach than traditional CNN-based systems reported in the literature. In addition, when specifically comparing our results with previously published chestnut classification studies, further performance advantages become evident. Recent research using classical machine learning techniques—such as feature extraction combined with SVM classifiers—reported moderate classification performance for chestnut phenotype or quality assessment^58,59^. Although these approaches demonstrated the feasibility of automated chestnut inspection, their dependence on hand-crafted features and limited capacity for capturing complex visual patterns constrained their accuracy and robustness. By contrast, the ViT-based model presented in this study leverages global attention mechanisms and learns discriminative representations directly from RGB images, enabling substantially improved multi-class classification accuracy without requiring specialized imaging systems. This highlights the contribution of our deep learning–based framework as a more advanced and scalable solution compared with traditional chestnut classification approaches documented in the literature. In future study, attention visualization of ViT which can represents how the classification results is extracted from deep learning models by uding Grad-CAM for CNNs.

The *k*-means clustering-ViT-based model to classify size, cultivar, and spoilage of chestnuts have significant potential of AI-driven image analysis for revolutionizing chestnut post-harvest handling. Automating the classification of chestnuts by size, cultivar, and spoilage offers substantial improvements over traditional manual sorting, leading to enhanced efficiency and reduced labor costs in the agricultural sector. First, automated size classification is crucial for standardizing product packaging and pricing, ensuring consistency for market demands and improving consumer satisfaction^8^. Accurate cultivar identification enables tailored post-harvest treatment and market differentiation, which is particularly valuable for maintaining the distinct characteristics of different chestnut varieties. Furthermore, the ability to sort spoilage effectively and early is paramount for minimizing post-harvest losses and ensuring food safety, directly impacting product shelf-life and consumer confidence. In addition, this finding also holds immense promise for improving peeling efficiency. By accurately classifying chestnuts based on their size and curtivar, which are key factors influencing optimal peeling conditions, our developed model can inform and optimize automated peeling processes^60,61^. This leads to reduced breakage, minimized material loss, higher yield of perfectly peeled chestnuts, ultimately contributing to increased profitability and reduced waste in processing facilities.

However, implementing the developed model for chestnut classification in real-world settings faces several key challenges^4,62^. Data quality and acquisition can be difficult due to variations in real-word environments (lighting conditions such as brightness, dirt, chestnut orientation such as rotation, image degradation such as noise etc.), potentially impacting model accuracy. Significant initial investment and technical effort are required for hardware and infrastructure setup, including selecting appropriate cameras and sensors for high-speed processing and ensuring seamless integration with existing equipment. In terms of model performance, issues like false positives and negatives could lead to substantial losses, making real-time processing speed and the model’s ability to generalize to new cultivars and conditions crucial. Finally, the high initial cost, ongoing maintenance, and the need for operator acceptance of automation are all critical hurdles that must be overcome for successful real-world deployment. Going forward, by applying our developed model, these challenges can be addressed through continued improvements in data augmentation techniques and closer collaboration with industry to refine system design for practical implementation. In addition, the number of acquired sample for rottenness is relatively small as compare to other classes. While evaluating the performance of deep learning model, class-weighted F1, PR-AUC curve need to be considered in future study. As the agricultural sector increasingly embraces digital transformation, the high initial cost, ongoing maintenance, and operator acceptance are also expected to become less of a hurdle over time, paving the way for the successful deployment of AI-based chestnut classification systems.

## Conclusions

The classification of chestnut physical characteristics is essential for large-scale commercialization, as it directly affects processing efficiency, product quality, and economic value. Traditional manual sorting remains labor intensive, time consuming, and prone to inconsistency, underscoring the need for automated vision-based solutions. In this study, four k-means clustering–deep learning models, including a Vision Transformer (ViT) and three CNN architectures, were developed to classify chestnuts into five cultivars, two size grades, and two rottenness states using a single-view RGB image. Among all models, the ViT consistently achieved the highest accuracy, precision, recall, and F1-score, demonstrating superior capability in recognizing subtle visual patterns compared to CNN-based approaches. These findings are consistent with recent literature reporting strong performance of ViT models in agricultural and food image classification tasks. Studies such as Panchbhai et al.^12,13^. and VijayaKumari et al.^52^. have shown the potential of lightweight CNNs, while more recent works^27,53,55^ highlight the advantages of ViT in capturing global dependencies and improving robustness under complex visual conditions. Our results further reinforce this trend, indicating that ViT-based frameworks represent a promising direction for practical, high-accuracy agricultural sorting systems. While the results are promising, several considerations should be noted to contextualize the scope of the present work and guide future research directions. The dataset was collected under controlled laboratory conditions with fixed lighting, a uniform background, and a single-view imaging setup. Such conditions help ensure data consistency but may not fully reflect the variability typically encountered in industrial sorting environments, where factors such as illumination changes, surface contamination, and diverse object orientations are common. The dataset al.so includes only five cultivars and a naturally limited number of rotten samples, suggesting opportunities to further evaluate the model’s generalization to broader production scenarios, rare defect types, and new cultivars. Moreover, because the approach relies on single-view RGB imaging, internal or partially occluded decay may not always be detectable, indicating that multi-view or multimodal sensing could enhance early defect identification. Although the proposed model shows strong potential for automated classification, real-time performance in high-throughput sorting lines has not yet been assessed. Hardware variability, domain shifts, and long-term model stability are additional aspects that warrant further investigation to support future deployment. Future studies could expand the dataset to include more diverse cultivars and production environments, incorporate multi-view or 3D imaging, and develop domain adaptation strategies to enhance robustness under varying conditions. Pilot-scale integration with industrial sorting hardware will be important for validating real-time applicability. Additional research directions—such as multi-label learning for simultaneous attribute prediction, defect severity estimation, temporal quality monitoring, and multimodal fusion using RGB-depth or hyperspectral imaging—may further strengthen early defect detection and overall grading performance. In conclusion, the proposed k-means clustering–ViT model provides an accurate, efficient, and scalable solution for automated chestnut classification and has strong potential to support intelligent postharvest management systems. As deep learning and vision technologies continue to advance, the approach demonstrated in this study may serve as a foundation for next-generation automated sorting solutions in the agricultural sector.

## Data Availability

The datasets used and/or analysed during the current study available from the corresponding author on reasonable request.

## References

[1] Li, R. et al. Nutritional biology of chestnuts: a perspective review. *Food Chem.* **395**, 133575 (2022). 35777207 10.1016/j.foodchem.2022.133575

[2] Takada, N. et al. Development of a portable chestnut sorting system using machine vision. *J. Jpn Soc. Agric. Mach.* **80**, 365–372 (2018).

[3] Corona, P. et al. Chestnut cultivar identification through the data fusion of sensory quality and FT-NIR spectral data. *Foods* **10**, 2575 (2021). 34828856 10.3390/foods10112575 PMC8618948

[4] Gold, M. A. et al. Competitive market analysis: chestnut producers. *HortTechnology* **16**, 360–369 (2006).

[5] Brosnan, T. & Sun, D. W. Inspection and grading of agricultural and food products by computer vision systems—a review. *Comput. Electron. Agric.* **36**, 193–213 (2002).

[6] Donis-González, I. R. *Microbial decay of fresh and peeled chestnuts and its control in Michigan*. ( Michigan State University, 2008).

[7] Moscetti, R. et al. Detection of mold-damaged chestnuts by near-infrared spectroscopy. *Postharvest Biol. Technol.* **93**, 83–90 (2014).

[8] Sivaranjani, A. et al. An overview of various computer vision-based grading system for agricultural products. *J. Hortic. Sci. Biotechnol.* **97**, 137–159 (2022).

[9] Zhu, L. et al. Deep learning and machine vision for food processing: a survey. *Curr. Res. Food Sci.* **4**, 233–249 (2021). 33937871 10.1016/j.crfs.2021.03.009 PMC8079277

[10] Kaur, S. et al. Computer vision-based tomato grading and sorting. In *Advances in Data and Information Sciences*. 75–84 (Springer, 2018).

[11] Tillett, R. D. *Image Analysis for Agricultural Processes*. (University of Wales, 1993).

[12] Hassankhani, R. & Navid, H. Potato sorting based on size and color in machine vision system. *J. Agric. Sci.* **4**, 235 (2012).

[13] Makky, M. & Soni, P. Development of an automatic grading machine for oil palm fresh fruit bunches based on machine vision. *Comput. Electron. Agric.* **93**, 129–139 (2013).

[14] Panchbhai, K. G. et al. Small-size CNN and modified MobileNetV2 to identify cashew nut and fruit diseases. *Multimed Tools Appl.* **83**, 89871–89891 (2024).

[15] Panchbhai, K. G. et al. Modified MobileNet with leaky ReLU and LSTM for soil type classification. *Earth Sci. Inf.* **18**, 77 (2025).

[16] Panchbhai, K. G. & Lanjewar, M. G. Enhancement of tea leaf disease identification using modified SOTA models. *Neural Comput. Appl.* **37**, 2435–2453 (2025).

[17] Uyar, K. et al. ABC-based weighted voting deep ensemble learning for multiple eye disease detection. *Biomed. Signal. Process. Control*. **96**, 106617 (2024).

[18] Yurdakul, M. et al. Almond varieties classification with genetic designed lightweight CNN architecture. *Eur. Food Res. Technol.* **250**, 2625–2638 (2024).

[19] Yurdakul, M. et al. MaxGlaViT: a lightweight vision transformer for glaucoma diagnosis. *Int. J. Imaging Syst. Technol.* **35**, e70159 (2025).

[20] Yurdakul, M. & Taşdemir, Ş. BC-YOLO: MBConv-ECA-based YOLO framework for blood cell detection. *Signal. Image Video Process.* **19**, 712 (2025).

[21] Guo, X. et al. Long-term quality retention and decay Inhibition of chestnut using thymol-loaded Chitosan nanoparticles. *Food Chem.* **374**, 131781 (2022). 34896943 10.1016/j.foodchem.2021.131781

[22] Pereira, G. A. & Hussain, M. A review of transformer-based models for computer vision tasks. *ArXiv* (2024).

[23] Lahoti, A. et al. Role of locality and weight sharing in image-based tasks. *ArXiv* (2024).

[24] Lu, B. et al. Recent advances in hyperspectral imaging technology in agriculture. *Remote Sens.* **12**, 2659 (2020).

[25] Amjoud, A. B. & Amrouch, M. Object detection using deep learning and vision transformers: a review. *IEEE Access.* **11**, 35479–35516 (2023).

[26] Islam, K. Recent advances in vision transformers. *ArXiv* (2022).

[27] Dosovitskiy, A. An image is worth 16×16 words: Transformers for image recognition. *ArXiv* (2020).

[28] Steiner, A. et al. How to train your ViT. *ArXiv* (2021).

[29] Maurício, J. et al. Comparing vision Transformers and CNNs for image classification. *Appl. Sci.* **13**, 5521 (2023).

[30] Deng, J. et al. Deep-learning-based wireless visual sensor system for Shiitake mushroom sorting. *Sensors* **22**, 4606 (2022). 35746391 10.3390/s22124606 PMC9231019

[31] Zheng, H. et al. Identifying strawberry appearance quality by vision Transformers. *J. Food Process. Eng.* **45**, e14132 (2022).

[32] Raghu, M. et al. Do vision Transformers see like cnns? *Adv. Neural Inf. Process. Syst.* **34**, 12116–12128 (2021).

[33] Khan, A. et al. A survey of vision Transformers and CNN–transformer variants. *Artif. Intell. Rev.* **56**, 2917–2970 (2023).

[34] Jubayer, F. et al. Detection of mold on food surfaces using YOLOv5. *Curr. Res. Food Sci.* **4**, 724–728 (2021). 34712960 10.1016/j.crfs.2021.10.003 PMC8529025

[35] Dai, G. et al. Improving YOLOv5 for sprouted potato detection. *IEEE Access.* **10**, 85416–85428 (2022).

[36] Alzubaidi, L. et al. Review of deep learning: concepts and applications. *J. Big Data*. **8**, 53 (2021). 33816053 10.1186/s40537-021-00444-8 PMC8010506

[37] Tan, M. & Le, Q. EfficientNet. *Proc. Int. Conf. Mach. Learn.*, 6105–6114 (2019).

[38] He, K. et al. Deep residual learning for image recognition. In *Proc. IEEE Conf. Comput. Vis. Pattern Recognit.* 770–778 (2016).

[39] Redmon, J. & Farhadi, A. YOLOv3: an incremental improvement. *ArXiv* (2018).

[40] Ikechukwu, A. V. et al. ResNet-50 vs VGG-19 for pneumonia classification. *Glob. Transit. Proc.* **2**, 375–381 (2021).

[41] Behera, S. K. et al. Deep fine-KNN classification of ovarian cancer. *J. Cancer Res. Clin. Oncol.* **150**, 361 (2024). 39052091 10.1007/s00432-024-05879-z PMC11272718

[42] Al-Jabbar, M. et al. Ebola optimization with modified DarkNet-53. *Alexandria Eng. J.* **75**, 29–40 (2023).

[43] Poornam, S. & Angelina, J. J. VITALT: vision transformer for brain tumor detection. *Neural Comput. Appl.* **36**, 6403–6419 (2024).

[44] Naidu, G. et al. Springer,. Review of evaluation metrics in machine learning. *Comput. Sci. On-line Conf.* 15–25 (2023).

[45] Joo, S. et al. Comparison of chestnut quality by storage temperature and cultivar. *J. Korean Soc. Sci.* **105**, 93–102 (2016).

[46] Pereira-Lorenzo, S. et al. Chestnut. In *Fruit Breeding* 729–769 (Springer,(2012).

[47] Seo, Y. C. A chestnut separator. *Korean Patent* 3010748980000 (2020).

[48] Wang, W. et al. Recognition of worm-eaten chestnuts by machine vision. *Math. Comput. Model.* **54**, 888–894 (2011).

[49] Guo, J. et al. CMT: CNNs meet vision transformers. In *Proc. IEEE/CVF Conf. Comput. Vis. Pattern Recognit.*, 12175–12185 (2022).

[50] Wang, X. et al. Chestnut shell polyphenols inhibit food-spoilage bacteria. *Foods* **12**, 3312 (2023). 37685244 10.3390/foods12173312 PMC10486611

[51] Webber, J. B. et al. Postharvest spoilage incidence in Chinese chestnut. *HortTechnology* **32**, 164–171 (2022).

[52] El Amin, G. M. et al. Smart food sorting using deep learning. In *Proc. Int. Conf. Adv. Electr. Eng.*, 1–5 (IEEE, 2024).

[53] Nasiri, A. et al. Automatic sorting system for unwashed eggs using deep learning. *J. Food Eng.* **283**, 110036 (2020).

[54] VijayaKumari, G. et al. Food classification using transfer learning. *Glob Transit. Proc.* **3**, 225–229 (2022).

[55] Gao, X. et al. High-accuracy food image classification via vision transformer. *J. Food Eng.* **365**, 111833 (2024).

[56] Xiao, Z. et al. CNN–transformer combination for medical image segmentation. *Comput. Methods Programs Biomed.* **226**, 107099 (2022). 36116398 10.1016/j.cmpb.2022.107099

[57] Xiao, Z. et al. Fine-grained food image recognition using Swin transformer. *J. Food Eng.* **365**, 112134 (2024).

[58] Yurdakul, M. et al. Chestnut varieties classification using HHO-selected features. In *Proc. iCACCESS*, 1–5 (IEEE, 2024).

[59] Yurdakul, M. et al. Webserver-based mobile application for chestnut classification. *Appl. Fruit Sci.* **67**, 102 (2025).

[60] Kim, T. H. et al. Machine learning clustering for analysis of chestnut peeling rate. *J. Korea Acad. -Ind Coop. Soc.* **25**, 1–7 (2024).

[61] Kim, T. H. et al. Prediction of weight loss rate of chestnut in knife peeling. *J. Korea Inst. Inf. Electron. Commun. Technol.* **17**, 236–244 (2024).

[62] Sofu, M. M. et al. Design of an automatic Apple sorting system using machine vision. *Comput. Electron. Agric.* **127**, 395–405 (2016).

